# Layer pullet preferences for light colors of light-emitting diodes

**DOI:** 10.1017/S1751731118002537

**Published:** 2018-10-12

**Authors:** G. Li, B. Li, Y. Zhao, Z. Shi, Y. Liu, W. Zheng

**Affiliations:** 1 Department of Agricultural Structure and Bioenvironmental Engineering, College of Water Resources and Civil Engineering, China Agricultural University, Beijing 100083, China; 2 Key Laboratory of Agricultural Engineering in Structure and Environment, Ministry of Agriculture, Beijing 100083, China; 3 Department of Agricultural and Biological Engineering, Mississippi State University, Mississippi, MS 39762, USA; 4 Beijing Engineering Research Center for Animal Health Environment, Beijing 100083, China

**Keywords:** poultry, light choices, monochromatic light, behaviors, test system

## Abstract

Light colors may affect poultry behaviors, well-being and performance. However, preferences of layer pullets for light colors are not fully understood. This study was conducted to investigate the pullet preferences for four light-emitting diode colors, including white, red, green and blue, in a lighting preference test system. The system contained four identical compartments each provided with a respective light color. The pullets were able to move freely between the adjacent compartments. A total of three groups of 20 Chinese domestic Jingfen layer pullets (54 to 82 days of age) were used for the test. Pullet behaviors were continuously recorded and summarized for each light color/compartment into daily time spent (DTS), daily percentage of time spent (DPTS), daily times of visit (DTV), duration per visit, daily feed intake (DFI), daily feeding time (DFT), feeding rate (FR), distribution of pullet occupancy and hourly time spent. The results showed that the DTS (h/pullet·per day) were 3.9±0.4 under white, 1.4±0.3 under red, 2.2±0.3 under green and 4.5±0.4 under blue light, respectively. The DTS corresponded to 11.7% to 37.6% DPTS in 12-h lighting periods. The DTV (times/pullet·per day) were 84±5 under white, 48±10 under red, 88±10 under green and 94±8 under blue light. Each visit lasted 1.5 to 3.2 min. The DFI (g/pullet·per day) were 27.6±1.7 under white, 7.1±1.6 under red, 15.1±1.1 under green and 23.1±2.0 under blue light. The DFT was 0.18 to 0.65 h/pullet·per day and the FR was 0.57 to 0.75 g/min. For most of the time during the lighting periods, six to 10 birds stayed under white, and one to five birds stayed under red, green and blue light. Pullets preferred to stay under blue light when the light was on and under white light 4 h before the light off. Overall, pullets preferred blue light the most and red light the least. These findings substantiate the preferences of layer pullets for light colors, providing insights for use in the management of light-emitting diode colors to meet pullet needs.

## Implications

In commercial layer pullet production, the use of light colors are primarily based on human perception, which may not reflect the biological or physiological needs of pullets and can therefore affect their well-being. Appropriate lighting management before the laying stage is critical to obtain good quality pullets, which can achieve their optimal genetic potential. Assessment of pullets’ choices among different light colors will help to determine the actual light needs of layer pullets, and be beneficial for implementation of optimal lighting practices for improving animal welfare and production efficiency.

## Introduction

Light is an important stimulus that the domestic fowl perceives from the physical environment (Rierson, 2011). Light management has profound effects upon the production efficiency, physiological and behavioral response of poultry (Manser, 1996). Recently, a more energy-efficient and durable light-emitting diode (LED) light is increasingly being applied in poultry production. The light spectrum and apparent color of LED can be manipulated more precisely by altering the chemical components in the LED as compared to traditional light sources (fluorescent or incandescent lights) that rely on filters for changing colors.

Research on the effect of various light colors on poultry has been conducted previously. It has been demonstrated that red light, as a long-wavelength radiation, can pass through hypothalamic extra-retinal photoreceptors and stimulate the reproductive axis (Lewis and Morris, 2000), thereby having an accelerating effect on activity stimulation, sexual development and maturity of poultry (Baxter *et al*., 2014; Li *et al*., 2014). Birds raised under a red light were more active and performed more walking and peaking behaviors (Sultana *et al*., 2013). Blue and green lights were found to be associated with improved growth, calm birds and enhanced immune responses (Cao *et al*., 2008; Sultana *et al*., 2013). Birds in the abovementioned investigations were under fixed monochromatic or mixed LED lights based on human perceptions, and these may not reflect the lighting needs of poultry.

Preference tests offer solutions to understand the environmental requirements from the animal’s standpoint (Dawkins, 1999). Senaratna *et al*. (2012) investigated the preferences of broilers for four light colors (red, green, blue and white) in three sessions of the day, and they found that the broiler preferred red light within 2 h after the light was turned on under tropical conditions. Rierson (2011) offered either pelleted or crumbled feed under four different light colors (red, white, blue and green) and concluded that broilers statistically preferred white lighting, followed by red, while green and blue lights were not statistically different. However, the abovementioned studies focused on broilers rather than layer pullets that may have different preferences for light colors.

Proper light management for layer pullets is critical as it affects their production performance at the laying stage (Hy-Line International, 2013). Assessment of light color preferences for layer pullets helps to understand the real light needs of pullets, thus having critical economic and welfare implications for poultry industry. The objective of this study was to investigate the preferences for different LED light colors by layer pullets by providing the birds with free choices of four light colors, including white, red, green and blue, in a lighting preference test system (LPTS).

## Material and methods

### Lighting preference test system

**Figure 1:**
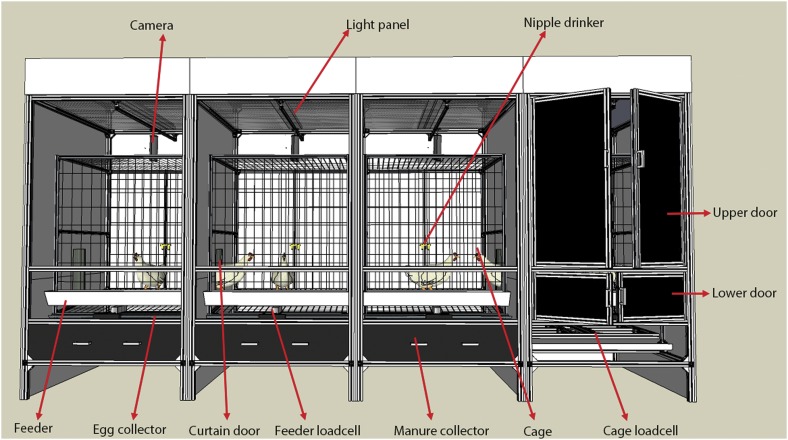
Schematic drawing of the lighting preference test system provided for layer pullets during the preference test. Note: every chamber has the upper and lower doors; four light colors (white, red, green and blue) are placed in four compartments.

The experiment was conducted in a LPTS (3.84 ml×1.2 mW×2.0 mH), which contained four identically individual compartments (0.96 ml×1.2 mW×2.0 mH) (Figure 1). Each compartment consisted of an aluminum cage (0.85 ml×0.85 mW×1.2 mH), a cage loadcell, a trough feeder, a feeder loadcell, a manure collector, an egg collector and nipple drinkers. A total of two pairs of double front doors were installed for daily management (e.g. egg collection, manure removal, mortality removal and system cleaning). The cage loadcell (50±0.0084 kg, MT1241; Mettler-Toledo International Inc., Changzhou, China) and feeder loadcell (7±0.001 kg, MT1022; Mettler-Toledo International Inc.) continuously detected the weight in the cage and feeder, respectively. Birds could freely move between two adjacent compartments/lighting conditions through a curtain door. The system had great light tightness based on previous validation (Li *et al*., 2018).

### Lighting environment

**Figure 2:**
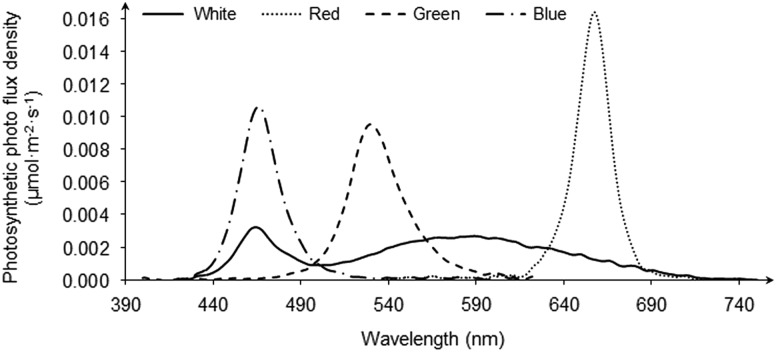
Light spectral distributions of four light-emitting diode (LED) lights (white, red, green and blue) at the intensity of 0.1 watt/m^2^.

An LED lighting control system was used for automatically controlling light colors, light intensities and light programs in this study. The system consisted of four lighting panels, a controller, a power cord and cables. The schematic drawing of the lighting control system is given in the Supplementary Figure S1. Each light panel was placed on the top of cage in each compartment and contained four LED channels, which were white (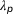
=464 and 600 nm), red (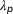
=656 nm), green (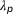
=530 nm) and blue (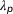
=466 nm). Each channel was packaged with desired light intensities and light programs and could be switched automatically among four compartments. Light intensities were specified by increasing/decreasing voltages. A timer was built into the controller to precisely turn on/off lights of each compartment. The spectrum distributions of these light colors are shown in Figure 2. The irradiance of each light color was 0.1 watt/m^2^ at bird head level (Rozenboim *et al*., 2004) as measured by a LED Grow Light Spectrometer (SRI-PL-6000+; Optimum Optoelectronics Corp., Chubei City, Taiwan). The irradiance of each light color, measured in 5-nm intervals, was converted to the relative photon flux of energy per unit wavelength. This was then multiplied by the known spectral sensitivity of the chicken according to Prescott and Wathes (1999) and summed to give the relative illuminance of the light colors for the chicken that is referred to as chicken lux, or clux. As in the Supplementary Table S1 shown, the chicken-perceived light intensities were 36.9±0.2 clux for white, 14.2±0.3 clux for red, 42.3±0.2 clux for green and 33.8±0.1 clux for blue light. The light program was a constant schedule of 12L:12D (lights on at 8:00 and lights off at 20:00 h).

### Animal and acclimation

In all, three batches of 20 50-day-old Chinese domestic layer pullets (Jingfen; Beijing Huadu Yukou Poultry Co. Ltd., Beijing, China) with white-color plumage were used for the preference test. The environmental managements of the pullets at 0 to 49 days of age followed industrial recommendations, and details are provided in the Supplementary Table S2. Upon arrival at the laboratory, the 20 pullets of each batch were kept in the LPTS to acclimate to the new lighting environment for 4 days. The sequence of light colors for acclimation was based on a 4×4 Latin Square arrangement. The arrangement of the light color sequence for the acclimation is given in the Supplementary Table S3. The curtain door was fully open on the first acclimation day, then the curtain strips were gradually dropped down in the next 4 days (1/4 of curtain strip per day). Then, the birds were allowed to move freely among various compartments to experience the experimental lighting conditions. After the acclimation, the 54-day-old birds started to present preferences on different lighting environments. Feeding and egg collection were performed at 0730 h every day. Manure was removed every 3 days.

### Preference test

After acclimation, the 20 birds were randomly distributed in four compartments. The assignment of four light colors followed another 4×4 Latin Square design (Table 1) in each test period, so each compartment contained all four light colors during the four periods. This was to prevent any compartment preferences to affect the choices of poultry (Ma *et al*., 2016). Temperature and relative humidity were maintained at 23.1±0.5°C and 20±1% during the experiment period. When 20 pullets were present together in a compartment, the stocking density was 361 cm^2^/pullet, which was higher than that recommended by Hy-Line International (2013) for cage-reared pullets (100 to 200 cm^2^/pullet).

**Table 1:** Light colors in each compartment (C1 to C4) provided for layer pullets during the preference test

Days of age	C1	C2	C3	C4
54 to 60	Red	Blue	White	Green
61 to 68	White	Red	Green	Blue
69 to 75	Green	White	Blue	Red
76 to 82	Blue	Green	Red	White

C1 to C4 represent the four compartments used in the preference test.

### Determination of pullet occupancy in compartments

The weights of the cages and feeders were continuously monitored and stored at 1-s intervals in the LabVIEW-based DAQ. Data were exported as CSV files from the DAQ system, then analyzed using visual basic application in Excel 2013. The number of pullets was determined using weight data as follows: overall BW in the four testing compartments (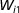, 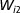, 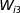 and 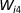) for every second was divided by 20 to get the instantaneous average weight per pullet (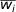). The number of pullets (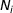) in a compartment was determined by dividing total pullet weight in that compartment with the average weight (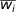) and then rounding to the nearest integer. The accuracy of the program on the recognition of pullet number was validated previously by human observation of the image and the agreement was 99% or better (Li *et al*., 2018).(1)
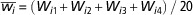

(2)
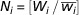



### Behavioral responses

Time spent is the overall time a pullet spends in a compartment during the lighting period (daily time spent (DTS)) or within each lighting hour (hourly time spent). Daily percentage of time spent (DPTS) is the percentage of time a pullet spends in a compartment during lighting hours. Daily times of visit (DTV) is the frequency of visit a pullet pays to a compartment within lighting period. Duration per visit (DV) is the time a pullet spends in a compartment during a single visit. Distribution of pullet occupancy (DPO) is the percentage of all the birds occupying a given compartment.

A feeder loadcell was installed underneath the feeder tough in each compartment, and the weights of the feeder were used to calculate feeding behavior. Daily feed intake (DFI) was calculated by comparing the difference in feeder weight at 0700 *v*. 2100 h (Table 2). The resolution of the scale for feeder weight was 2 g. Daily feeding time (DFT) was the time pullets spend at feeder in the detected feeding events. Feeding rate (FR) is calculated by dividing DFI with DFT.

**Table 2:** The behavioral parameters of layer pullets measured during the preference test

Parameter	Unit	Definition
Daily time spent (DTS)	h/pullet·per day	Overall time spent in a compartment with a specific light color within a day
Daily percentage of time spent (DPTS)	%	DTS/12 h×100%
Daily times of visit (DTV)	times/pullet·per day	Number of visits to a compartment within a day
Duration per visit (DV)	Min/visit	DTS/DTV
Daily feed intake (DFI)	g/pullet·per day	Feed consumption within a day
Daily feeding time (DFT)	h/pullet·per day	Overall time spent at feeder within a day
Feeding rate (FR)	g/min	DFI/DFT
Distribution of pullet occupancy (DPO)	%	Percentage of birds in a compartment
Hourly time spent in compartment (HTS)	Min/pullet·per hour	Time spent in a compartment within each hour within a day

### Statistical analysis of results

The first 4 days of each test period were considered as acclimation periods, thus only data from the last 3 days of testing were used for data analysis. Therefore, a total of 12 days of data in each test period were included in the statistical analysis. All data were analyzed using PROC GLM statement in Statistical Analysis Software (SAS 9.3; SAS Institute Inc.). More details of the GLM procedure are described in the Supplementary Material S1. The differences between treatment means were examined by including light colors, compartments and their interactions. Effects of the light colors on the preference choices of layer pullets were compared by adopting a Tukey statement. Effects were considered significant when *P*<0.05. A sample of code used for data analysis is given in the Supplementary Material S2.

## Results

### Time spent under different light colors

Light colors had a significant effect on DTS and DPTS, whereas compartments and interactions between light colors and compartments had no significant effect (Table 3). Pullets stayed significantly longer under blue light than they did under white and green lights, and least under red light (*P*<0.05).

**Table 3:** Distribution of behavioral responses of layer pullets provided with free choice among four light colors (white, red, green and blue)

	Light colors					
Parameter	White	Red	Green	Blue	*P*-value	RMSE
DTS (h/pullet·per day)	3.9±0.4^b^	1.4±0.3^d^	2.2±0.3^c^	4.5±0.4^a^	<0.0001	0.44
DPTS (%)	32.4±2.9^b^	11.7±2.3^d^	18.3±2.1^c^	37.6±3.0^a^	<0.0001	3.70
DTV (times/pullet·per day)	84±5^a^	48±10^b^	88±10^a^	94±8^a^	<0.0001	13.40
DV (min/visit)	2.8±0.2^a^	2.0±0.2^b^	1.5±0.1^c^	3.2±0.3^a^	<0.0001	0.40
DFI (g/pullet·per day)	27.6±1.7^a^	7.1±1.6^c^	15.1±1.1^b^	23.1±2.0^a^	<0.0001	3.19
DFT (h/pullet·per day)	0.65±0.23^a^	0.18±0.08^d^	0.39±0.10^c^	0.57±0.13^b^	<0.0001	0.06
FR (g/min)	0.75±0.17^a^	0.57±0.33^b^	0.67±0.17^ab^	0.69±0.19^ab^	0.02	0.11

DTS=daily time spent; DPTS=daily percentage of time spent; DTV=daily times of visit; DV=duration per visit; DFI=daily feed intake; DFT=daily feeding time; FR=feeding rate. RMSE means the root mean square error. Each value is the mean of 36 observations. ^a,b,c,d^Values within a row with different superscripts differ significantly at *P*<0.05 (PROC GLM, LSD test).

### Visit to different light colors

Light colors had a significant effect on DTV and DV, whereas compartments and the interactions between light colors and compartment had no significant effect (Table 3). Visits to the compartments with red light were significantly fewer than to the compartments with other light colors (*P*<0.05). The DV under white and under blue lights were not significantly different, the duration was longer than under red light (*P*<0.05) and the shortest visit duration was found under green light (*P*<0.05).

### Feeding behavior

Daily feed intake and FR were significantly affected by light colors, but not by compartments or the interactions between light colors and compartments (Table 3). Daily feeding time was significantly affected by light colors, compartments and their interactions (*P*<0.05). The DFI was not significantly different between white and blue and was the least under red light (*P*<0.05). The DFT under white light was significantly higher than under blue and green lights, and DFT under red light was the shortest (*P*<0.05). A significant difference of FR was only detected between white and red light (*P*<0.05; Table 3).

### Distribution of pullet occupancy

**Figure 3:**
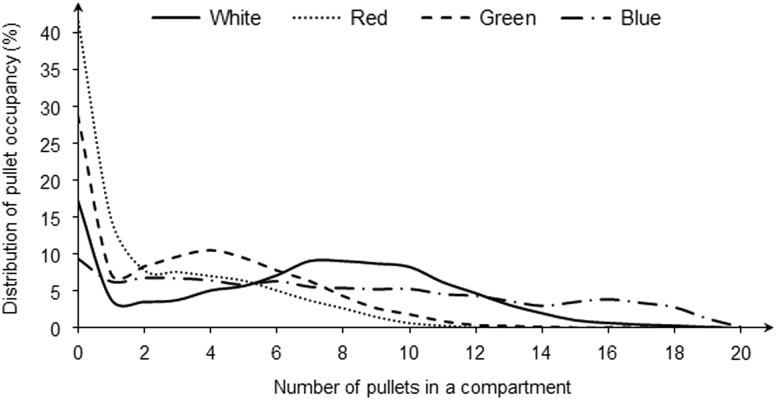
Distribution of pullet occupancy between compartments under different light colors (white, red, green and blue).

Figure 3 shows the DPO under different light colors. In general, the scenarios of zero birds took the largest proportion among all treatments. Under white light, the distributions of one to five birds, six to 10 birds, 11 to 15 birds and 15 to 20 birds were 22.0%, 42.0%, 17.1% and 2.7%, respectively; under red light, the same distributions were 43.8%, 13.7%, 0.5% and <0.1%, respectively; under green light, the distributions were 45.5%, 23.1%, 2.0% and 0.6%, respectively; and under blue light, the distributions were 32.1%, 27.9%, 19.1% and 15.0%, respectively.

### Hourly time spent in a compartment

**Figure 4:**
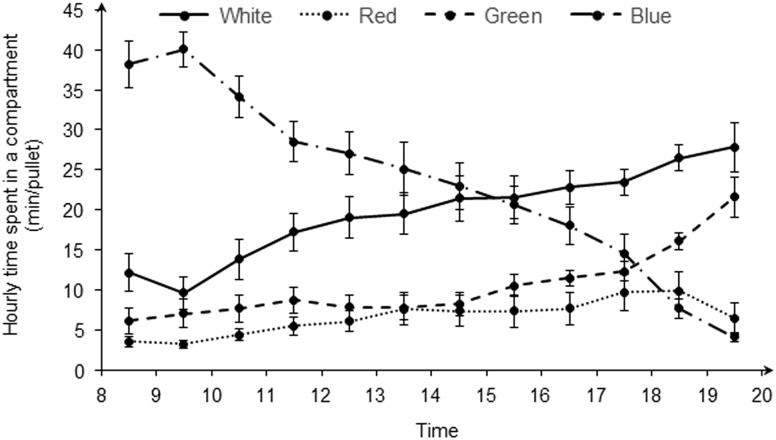
Hourly time spent of layer pullets in compartments under different light colors (white, red, green and blue).

Figure 4 shows the time pullet spent in different light colors within each lighting hour. For blue light and white light, pullets spent more time under blue light from 0800 to 1400 h than under white, and less time under blue than under white light from 1500 to 2000 h. The average time spent under blue or white light was more than that spent under green or red light. The time pullets spent in the compartment with green light increased from 0800 to 1800 h, and the average time spent under green light was more than that under red and less than that under white light. Interestingly, the time spent under green light was greater than that under blue light at 1700 h. Pullets spent the least amount of time (on average 5 min/h) under red light from 0800 to 1800 h.

## Discussion

### Hybrid and bird age

Jinfen layer pullet, a Chinese domestic layer strain, is a popular breeder chicken in China; however, the optimal environmental management is still not fully known as it is a relatively new strain compared with Hy-Line chicken. As public concern regarding animal welfare increases, the environmental management, including light colors, need to be explored and introduced based on animal requirements. According to data from Jinfen and Hy-Line International (2013), 7- to 12-week-old pullets are in the key period of bone, muscle and feather development. Blue/green light has been shown to improve the body development of pullets and red light can stimulate the sexual maturity of chickens (Lewis and Morris, 2000). However, production performance is only one aspect, and if these light colors are also preferred by the pullets, they would have both economic and welfare significance for the poultry industry.

### Time spent under different light colors

Pullets spent the most time under blue light and least under red light. These results were partially consistent with those of Prayitno *et al*. (1997), who reported that chickens preferred blue or green light over red or white light. Riber (2015) concluded that poultry preferred blue light or light containing a high-power emission from the blue part of spectrum. In the present study, pullets preferred blue light most and red light least. Based on the majority of previous studies, blue light can reduce aggressive tendencies of pullets and red light can stimulate aggressiveness (Lewis and Morris, 2000). The pullets secondly preferred white light presumably because its spectrum (between 400 and 750 nm) was relatively close to the sunlight spectrum, which is also preferred by birds (Gunnarsson *et al*., 2008).

Attention should be drawn to birds perceived light intensity differently from humans due to differences in spectral sensitivity (Prescott and Wathes, 1999). In the present study, we measured lights in watt/m^2^, which revealed differences in the perceived intensities among the four light colors for pullets (Supplementary Table S1). Davis *et al*. (1999) conducted preference tests under light intensities of 6, 20, 60 and 200 lux for broilers and laying hens at 2 and 6 weeks of age, and found that younger birds preferred the brightest lights whereas older birds preferred to be in dimmest light. Ma *et al*. (2016) investigated the light intensity preferences of laying hens at the intensities of <1, 5, 15, 30 and 100 lux and concluded that except for the darkness treatment (<1 lux), birds spent most time at the lower intensities of 5 and least time at the highest intensities of 100 lux. In the present study, pullets neither preferred brightest light (green) nor dimmest light (red). That might be affected by a number of factors, including light colors, bird ages and strain. It should also be noted that the main goal of this study was to evaluate the light color preferences rather than light intensity preferences. More independent experiments should be conducted to obtain more consistent results of light intensity preferences for poultry.

The birds had a significant preference for blue and white lights, but their behavior also implied that they spent time in some less preferable environments. The result was consistent with the results of Liu *et al*. (2017) and Rierson (2011), who reported that poultry spent time in all given lighting environments during the preference test, no matter if it was a preferable lighting environment or not. Previous research have proved that dominant hens had priority use of resources, for example feeders, nest box and dust bath, resulting in subordinate hens not being able to use the facilities fully (Weeks and Nicol, 2006; Shimmura *et al*., 2008). Specifically, in this experiment, birds with lower social hierarchy occasionally waited in a less desirable lighting environment until the birds with higher social hierarchy left the adjacent compartment, which may be part of the reason why poultry also spent time in less preferable environments.

### Visits to different light colors

Pullets visited the compartment with red light less than those with other light colors. This result adds to the evidence that red is the least preferable light color by pullets. The DTV were not significantly different among white, green and blue light, which indicated that pullet preferred to visit multiple light colors rather than continuously stay in a single lighting environment. This result is consistent with that of Ma *et al*. (2016), who also found that laying hens possibly like diverse lighting environments instead of an unchanged one. However, based on current data, it was difficult to discern if the DTV was caused by visiting preferred light colors or by just passing through. Pullet spent the same time under white and blue lights for a single visit, and less time under green and red lights. The assessment of DV could provide information on an animal’s motivation to exit or enter a specific environment (Kristensen *et al*., 2000), so the results of DV also support that the pullets preferred blue and white lights in this study.

### Feeding behavior

Pullet ate most under white and blue lights, and least under red light. Rierson (2011) conducted a preference test of light color on feed consumption of broilers and obtained similar observations that from weeks 4 to 6, the chicks preferred eating under white light. A possible explanation as to why pullets prefer eating under white light could be because it helps them identify the feed from an environment they cannot see under other light colors. More research needs to be conducted to investigate further this possibility.

Pullets spent more time feeding under white and least under red light. The results were partly in tune with that of Prayitno *et al*. (1997), who found that female birds increased feeding time under white and reduced it under red. However, Huber-Eicher *et al*. (2013) examined the effects of white, red and green LED on the feeding behavior of laying hens and found no significant difference for time spent feeding among light treatments. One thing should be noticed in this study was that DFT was affected by light colors, compartment and their interaction.

Pullets ate faster under white than they did under red light. There is a lack of comparative literature reporting the FR in preference tests of light colors for poultry. Ma *et al*. (2016) investigated the preferences of laying hens for different light intensities and reported a FR of ~0.4 g/min. The difference in FR between these two studies may be due to the day lengths (12 *v*. 24 h), light colors (red, green and blue *v*. white), light intensities (0.1 watt/m^2^ *v*. <1, 5, 15, 30 and 100 lux), bird ages (54 to 82 days of age *v*. 161 to 210 days of age) and bird breed (Jinfen layer pullets *v*. W-36 laying hens).

### Distribution of pullet occupancy

For most of the lighting period, six to 10 birds were in the compartment with white light, whereas one to five birds were in the compartments with red, green, and blue light, respectively. Clearly, the capacities of the compartments under various light colors, especially under red light, were not fully utilized. Compared to the standard of Hy-Line International (2013) that recommended 100 to 200 cm^2^/pullet (converted to 36 to 72 pullets/compartment in this case), the pullets were provided with more compartment space to avoid potential competition due to space limitations.

### Hourly time spent at a compartment

Pullets preferred to stay under blue light within 7 h after the light was turned on and under white light within 4 h before the light was off. The light was turned on abruptly in the present study, which may cause stress for pullets (Portocarero, 2011). Compared with other light colors, blue light could calm the birds (Sultana *et al*., 2013). This may be the reason pullets chose the blue light after the light was abruptly turned on. Gunnarsson *et al*. (2008) found that the layer pullets preferred natural light over incandescent light. In the present study, the light spectrum of the white light was more similar than the other three colors to the natural light, which may be why the time spent under white light gradually increased and peaked before the light was turned off.

## Conclusion

Preferences of layer pullets (8 to 12 weeks of age) to LED lights of white, red, green or blue was assessed using a LPTS. Pullets spent the longest time under blue light and the least time under red light. Specifically, they preferred to stay under blue light within 7 h after light on and under white light within 4 h before the light off. They visited the compartment with red light significantly less and ate more under white and blue lights. The results provide some insights into the management of light colors with regard to the pullets’ welfare.
