# Supplementary material for: Layer pullet preferences for light colors of light-emitting diodes
Source: Animal. 2018 Oct 12;13(6):1245–51. doi: 10.1017/S1751731118002537 (PMC6528386; doi:10.1017/S1751731118002537)
Supplement: Supplementary file 1 [file S1751731118002537sup001.docx]

Layer Pullet Preference for Light Colors of Light-Emitting Diode (LED)

Guoming Li, Baoming Li, Yang Zhao, Zhengxiang Shi, Yu Liu, and Weichao Zheng

**Supplementary material**

**Table S1** *Irradiance and light intensities (human-perceived and chicken-perceived) for the light colors used during the preference test*

| Light units | White | Red | Green | Blue |
| --- | --- | --- | --- | --- |
| Irradiance (watt·m^-2^) | 0.1±0.0 | 0.10±0.0 | 0.1±0.0 | 0.1±0.0 |
| Human-perceived light intensity (lux) | 24.8±0.1 | 5.4±0.1 | 40.1±0.1 | 6.1±0.1 |
| Chicken-perceived light intensity (clux) | 36.9±0.2 | 14.2±0.3 | 42.3±0.2 | 33.8±0.1 |

**Table S2** *Lighting and housing conditions of Jinfen layer pullets from 1 to 7 weeks of age*

| Age | | Lighting schedule (L:D) | Light intensity (lux) | Temperature (°C) | Relative humidity (%) |
| --- | --- | --- | --- | --- | --- |
| Days of age | 1-3 | 24L:0D | 60 | 36 | 60 |
|  | 4-7 | 22L:2D | 30 | 34 | 58 |
| Weeks of age | 2 | 20L:4D | 30 | 32 | 58 |
|  | 3 | 18L:6D | 30 | 30 | 58 |
|  | 4 | 16L:8D | 30 | 28 | 58 |
|  | 5 | 14L:10D | 30 | 26 | 58 |
|  | 6 | 12L:12D | 30 | 24 | 58 |
|  | 7 | 12L:12D | 30 | 22 | 58 |

**Table S3** *Light colors assigned to different compartments during the acclimation period*

| Day of age | C1 | C2 | C3 | C4 |
| --- | --- | --- | --- | --- |
| 50 | White | Red | Blue | Green |
| 51 | Red | White | Green | Blue |
| 52 | Blue | Green | White | Red |
| 53 | Green | Blue | Red | White |

C1-C4 in the table mean the numbers of compartment in the LPTS.


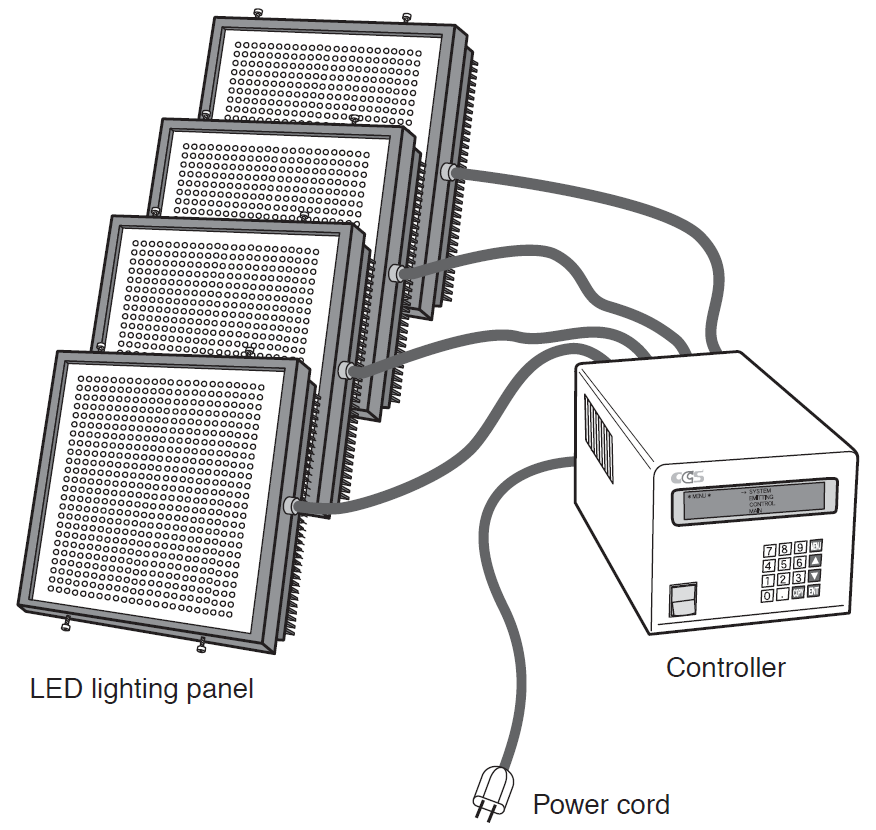


**Figure S1** Schematic drawing of light-emitting diode (LED) lighting control system

**Material S1** GLM procedure and model description

A two-way generalized linear model (GLM) procedure was introduced to analyze the light colors effect, compartment effect and their interaction on behavioral responses during the preference test. The model is defined as

$$Y_{ijk}=\mu+\alpha_{i}+\beta_{j}+{\alpha\beta}_{ij}+\varepsilon_{ijk}$$

Where

$Y_{ijk}$ is the measured behavioural responses during the preference test

$\mu$ is the overall mean

$\alpha_{i}$ is the main effect of light colors, $i=1, 2, 3, 4$

$\beta_{j}$ is the main effect of compartment, $j=1, 2, 3, 4$

${\alpha\beta}_{ij}$ is the interaction effect of light colors and compartment

$\varepsilon_{ijk}$ is the random error for the model

**Material S2** SAS code for daily time spent (DTS) in data analysis

**PROC** **IMPORT** OUT= WORK.Dailytimespent

DATAFILE= "path of data file"

DBMS=EXCEL REPLACE;

RANGE="DTS$";

GETNAMES=YES;

MIXED=NO;

SCANTEXT=YES;

USEDATE=YES;

SCANTIME=YES;

**RUN**;

**PROC** **GLM** DATA=Dailytimespent;

CLASS color compartment;

MODEL DTS=color compartment color*compartment;

MEANS color compartment color*compartment/TUKEY CLDIFF;

**RUN**;

**PROC** **MEANS** DATA=Dailytimespent MEAN STDERR;

CLASS color;

**RUN**;
